# Nanoliposome-Encapsulated Brinzolamide-hydropropyl-β-cyclodextrin Inclusion Complex: A Potential Therapeutic Ocular Drug-Delivery System

**DOI:** 10.3389/fphar.2018.00091

**Published:** 2018-02-13

**Authors:** Fazhan Wang, Xingting Bao, Aiping Fang, Huili Li, Yang Zhou, Yongmei Liu, Chunling Jiang, Jinhui Wu, Xiangrong Song

**Affiliations:** ^1^State Key Laboratory of Biotherapy, Geriatrics and Cancer Center, West China Hospital and Collaborative Innovation Center for Biotherapy, Sichuan University, Chengdu, China; ^2^West China School of Public Health, Sichuan University, Chengdu, China

**Keywords:** brinzolamide, cyclodextrin, inclusion complex, nanoliposomes, intraocular pressure, ocular drug delivery

## Abstract

Novel ocular drug delivery systems (NODDSs) remain to be explored to overcome the anatomical and physiological barriers of the eyes. This study was to encapsulate brinzolamide (BRZ)-hydropropyl-β-cyclodextrin (HP-β-CD) inclusion complex (HP-β-CD/BRZ) into nanoliposomes and investigate its potential as one of NODDS to improve BRZ local glaucomatous therapeutic effect. HP-β-CD/BRZ was firstly prepared to enhance the solubility of poorly water-soluble BRZ. The HP-β-CD/BRZ loaded nanoliposomes (BCL) were subsequently constructed by thin-film dispersion method. After the optimization of the ratio of BRZ to HP-β-CD, the optimal BCL showed an average size of 82.29 ± 6.20 nm, ζ potential of -3.57 ± 0.46 mV and entrapment efficiency (EE) of 92.50 ± 2.10% with nearly spherical in shape. The X-ray diffraction (XRD) confirmed the formation of HP-β-CD/BRZ and BCL. The *in vitro* release study of BCL was evaluated using the dialysis technique, and BCL showed moderate sustained release. BCL (1 mg/mL BRZ) showed a 9.36-fold increase in the apparent permeability coefficient and had a sustained and enhanced intraocular pressure reduction efficacy when compared with the commercially available formulation (BRZ-Sus) (10 mg/mL BRZ). In conclusion, BCL might have a promising future as a NODDS for glaucoma treatment.

## Introduction

Despite easy accessibility for topical drug administration, overcoming the anatomical and physiological barriers of the eye remains one of the greatest challenges for ocular drug delivery (Bucolo et al., 2012; Zhang et al., 2015). Drug retention is impeded by tear reflex, blinking, and nasolacrimal drainage (Morrison et al., 2013). Cornea barrier protects the eye from the passage of any foreign molecules including drugs into the eye, and thus only a small fraction of the topically applied drug penetrates the cornea and reaches intraocular tissues (Mun et al., 2014). As a result, repeated dosing is needed, which increases the patient discomfort and other adverse effects (Luo et al., 2011). A system which behaves like a solution and at the same time can lead to retention of drug in the eye and increase corneal permeability is of great urgency.

Nanoliposomes, as biodegradable, biocompatible and non-immunogenic drug delivery system, have been widely applied (van Rooijen and van Nieuwmegen, 1980; Mugabe et al., 2006; Arias, 2013; Sercombe et al., 2015; Ji et al., 2017). They can encapsulate hydrophilic drugs into the inner aqueous phase, whereas entrap hydrophobic drugs into the hydrophobic lipid phase (Allen and Cullis, 2004; Chen et al., 2009). However, it is difficult to obtain high drug-loading capacity in the lipid phase because of the limited space offered by the lipid phase, and a large amount of hydrophobic drugs can destabilize the structural integrity of the liposomal layers (Song et al., 2012). Inclusion complexes are one of the most useful strategies in the pharmaceutical field, which can solubilize a wide range of hydrophobic drugs (Morrison et al., 2013; Zhang et al., 2013; Alomrani et al., 2014; Ji et al., 2016; Maria et al., 2017; Soliman et al., 2017). The binding force between the guest drug molecule and the cyclodextrin host molecule is usually weak, possibly leading to rapid dissociation of the inclusion complexes due to dilution by plasma or extracellular fluids (Chen et al., 2014). Encapsulation of hydrophobic drugs in the form of inclusion complexes into nanoliposomes has been investigated as a new strategy to combine the advantages of inclusion complexes and nanoliposomes, namely the drug-in-cyclodextrin-in-nanoliposome (DCL) system (Piel et al., 2006; Hatzi et al., 2007; Zhang et al., 2015). The presence of cyclodextrin in the aqueous compartment of nanoliposomes would not affect the characteristics of conventional liposomes but prolong drug release compared to conventional liposomes (Maestrelli et al., 2010; Zhang et al., 2015). Although a number of studies on DCL systems have been reported (Dhule et al., 2012; Rahman et al., 2012; Lapenda et al., 2013; Alomrani et al., 2014), to date, the therapeutic application of DCL systems as an ocular delivery system remains to be explored.

Nanoliposome systems are applicable in ocular delivery, which have the potential of increasing corneal permeability and improving retention time (de Sa et al., 2015; Li et al., 2016). Moreover, some functional liposomes have been constructed (Eriksen et al., 2017; Huang et al., 2017; Tan et al., 2017). Cyclodextrin, with a hydrophilic outer surface and a cavity at its center and low renal toxicity, has the ability to form hydrophilic inclusion complexes by a molecular complexation with a lot of hydrophobic drugs (Ji et al., 2016; Maria et al., 2016, 2017; Abd El-Gawad et al., 2017). Hydropropyl-β-cyclodextrin (HP-β-CD), a type of cyclodextrin, is commonly used for ocular drug delivery and has been approved by the FDA (Bozkir et al., 2012). Many *in vitro* and *in vivo* studies also showed that the inclusion complex of poor water-soluble drugs by HP-β-CD could increase drug solubility, corneal permeability, and ophthalmic bioavailability (Ito et al., 2013; Morrison et al., 2013). Encouraged by these previous results, we postulated that DCL might have the potential to enhance ocular permeation of the hydrophobic model drug brinzolamide (BRZ) and enhance its therapeutic efficacy for glaucoma.

**FIGURE 1:**
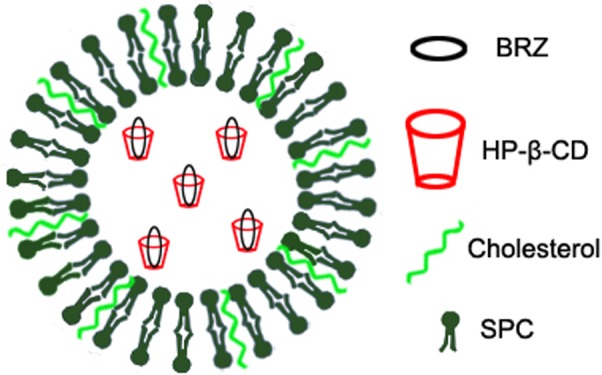
Schematic structure of HP-β-CD/BRZ-inclusion complex loaded nanoliposomes.

Previously, we have used inclusion complex and nanoliposomes to encapsulate the low water-soluble drug BRZ, respectively. Both formulations improved the corneal permeation and therapeutic efficacy of BRZ to some extent (Zhang et al., 2013; Li et al., 2016). Here, we further combined HP-β-CD and nanoliposomes to prepare BRZ-HP-β-CD inclusion complex (HP-β-CD/BRZ)-loaded nanoliposomes (BCL) as shown in **Figure 1**. The pharmaceutical properties including UV/vis spectra, particle size, surface charge, entrapment efficiency (EE) of BRZ, X-ray diffraction (XRD), and *in vitro* release profile were then characterized systemically. The *ex vivo* corneal permeability, *in vivo* intraocular pressure (IOP) reduction efficiency and preliminary safety of BCL were finally evaluated.

## Materials and Methods

### Materials

BRZ (purity > 99%) was obtained from Dalian Meilun Biology Technology Company (Dalian, China). HP-β-CD (purity > 99%, DS = 7) was supplied by Xi’an Deli Chemicals Corporation (Xi’an, China). Soybean phosphatidylcholine (SPC) was provided by Shanghai A.V.T. Pharmaceutical, Co., Ltd. (Shanghai, China). Cholesterol was purchased from Shanghai Yuanju Biology Technology Company (Shanghai, China). The commercial formulation AZOPT^®^ (BRZ-Sus) was supplied by Alcon Laboratories (Puurs, Belgium). All the other reagents were of analytical grade unless otherwise stated.

### Animals

White New Zealand rabbits (male, 2.0–2.5 kg) were used for each formulation (*n* = 6). Animals were housed individually and allowed free access to food and water with a 12:12 h cyclic lighting schedule. After a week of adaptation, animals were admitted to experiments. All animal experiments were approved and supervised by the State Key Laboratory of Biotherapy Animal Care and Use Committee (Sichuan University, Chengdu, Sichuan, China).

### Preparation of H-β-CD/BRZ Inclusion Complex

According to previously described methods by Zhang et al. (2013), the inclusion complex was prepared with some modifications. Briefly, BRZ and HP-β-CD at different molar ratio (2:1, 1:1, 1:2, and 1:3) were dissolved by ethanol in a round-bottomed flask. After 1 h of ultrasound at 37°C, the ethanol was removed using a rotary evaporator in vacuum. To dissolve the inclusion complex, PBS was added to the flask followed by filtering through a 0.22 μm membrane. The content of BRZ in the inclusion complex was analyzed by high-performance liquid chromatography (HPLC, Waters, Milford, MA, United States) at 252 nm. The mobile phase, at 1 mL/min flow rate, was composed of acetonitrile and water at a volume ratio of 40:60.

### Preparation of BCL

BCL was prepared using a modified thin-film dispersion method (Lapenda et al., 2013; Li et al., 2016). Briefly, SPC and cholesterol were dissolved in a mixture solvent of chloroform/methanol (4:1, v/v). The organic solvents were evaporated using a rotary evaporator at 37°C, and then the formed thin film was further dried under high vacuum for 1 h. The lipid film was hydrated with HP-β-CD/BRZ inclusion complexes at 60°C for 1 h to obtain a suspension. To obtain BCL, the above suspension was sonicated for 3 min at 100 W in an ice bath and filtered through a 0.22-μm membrane filter.

The BRZ-loaded nanoliposomes (LP/BRZ) were obtained using the similar procedure with the addition of BRZ into the chloroform/methanol (4:1 v/v) solvent mixture and hydrating lipid film with sterile PBS. The blank liposomes (Blank LP) were also prepared using the similar preparation process of LP/BRZ without adding BRZ.

### Characterization of BCL

#### Ultraviolet-Visible (UV/vis) Spectroscopy

The UV/vis absorption spectra of BRZ, HP-β-CD, Blank LP, inclusion complex, LP/BRZ, and BCL were recorded using a SHIMADZU UV-2600 UV/vis spectrophotometer in the range of 200–600 nm.

#### Particle Size and ζ Potential

The average particle size, size distribution [polydispersity index (PDI)], and ζ potential of diluted BCL were recorded by Zetasizer Nano ZS90 (Malvern Instruments, Malvern, United Kingdom). All measurements were carried out at 25°C after 3 min of equilibration and were conducted in triplicate. All the data were presented as mean ± standard deviation (SD).

#### Morphology

The morphological examination of BCL was performed by transmission electron microscopy (TEM, H-600, Hitachi, Japan). Briefly, a drop of liposomal suspension was placed onto copper electron microscopy grids, and then they were negatively stained with 2% phosphotungstic acid for observation at an acceleration voltage of 100 kV.

#### Drug Entrapment Efficiency

According to previously described methods (Rahman et al., 2012), the drug content of BCL was determined by separating BCL from the unentrapped drug using cooling centrifugation at 50,000 rpm for 30 min at a temperature of 4°C. Following the removal of the supernatant, 1 ml of acetonitrile was added into the BCL sediment and sonicated for 20 min to extract BRZ from BCL. After centrifugation at 13,000 rpm for 10 min, the content of BRZ in the supernatant diluted with acetonitrile was quantified by HPLC as described previously. Analysis was performed in triplicate and the values were expressed as mean ± SD. EE was calculated by the following calculation equation:

EE⁢= (amount of BRZ in BCL/initial BRZ amount)   x⁢  100%.

#### Stability of BCL

The optimized formulation was stored at 4°C for 2 weeks in order to investigate the preliminary stability. In the test, the physicochemical characteristics such as particle size and EE were recorded and changes over time were evaluated.

#### XRD Analysis

X-ray diffraction was performed to investigate the crystal structure according to the former study (Tao et al., 2017). Different samples (pure BRZ, HP-β-CD, HP-β-CD/BRZ, and BCL) were examined using an X-ray diffractometer (X’Pert Pro Philips, Netherlands) at a voltage of 40 kV and a current of 40 mA. The scans were carried out at a scanning rate of 10°C/min in the 2θ range from 0 to 50°C.

#### *In Vitro* BRZ Release from BCL

*In vitro* release studies were carried out using the dynamic dialysis technique in sink conditions. Briefly, 2 mL of different formulations containing the drug were placed into different dialysis bags (molecular weight cut-off = 3,500 Da) and immersed in 40 mL of simulated tear fluid (STF) and continuously agitated in an orbital shaker maintained at 37°C. At given time intervals, 1 mL of receptor phase was removed and replaced with an equal volume of STF. The drug concentration was quantified by the HPLC method as described above. All data were presented as mean ± SD of three independent measurements.

### Corneal Permeability

The transcorneal permeability of BCL was evaluated using a Franz diffusion chamber which consisted of a donor and a receiver compartment (with a volume of 1.0 and 2.0 mL, respectively). The diffusion chamber was maintained at a constant temperature (37 ± 0.2°C) by a thermostatic water bath, under mixing conditions using a magnetic stirrer. The cornea together with a 2-mm ring of sclera was excised immediately after the rabbits (*n* = 3) were sacrificed and stored in glutathione bicarbonate ringer (GBR) buffer after rinsing with cold saline before use. One mL of the GBR solution was added to the endothelial (receptor) side of the cornea, while 0.5 mL of different formulations was added to the epithelial (donor) side of the cornea. Different samples from the receptor chamber were withdrawn at 0, 0.5, 1, 1.5, 2, 3, 4, 5 and 6 h and replaced with fresh GBR buffer-receptor solution. The content of drug permeation across the cornea was determined by the HPLC method as described above. The experiment was done in triplicate.

The apparent permeation coefficient (P_app_, cm/s) of each formulation was calculated using the following equation (Goskonda et al., 2000):

$$P_{app}\,\;=\;\;\,\;{\left(1/AC_{0}\right)}^{\;*}\;\;\left(\mathrm{dM/dt}\right)$$

where A is the available corneal surface area for diffusion (cm^2^), C_0_ is the initial drug concentration (μg/mL) in the donor compartment, and dM/dt is the flux across the cornea (μg/cm^2^h).

### Corneal Hydration Level

According to the literature (Vega et al., 2008), the corneal hydration level (HL) was evaluated by measuring total water content by the gravimetric method. At the end of the corneal permeability study (*n* = 3), the wet weight of each cornea (W_1_) and dry corneal weight (W_2_) after a desiccation at 100°C for 6 h were obtained, respectively. The corneal HL was calculated using the following equation: HL% = (W_1_-W_2_)/W_1_ × 100%.

### *In Vivo* IOP Measurement

IOP was recorded in mmHg with a Tono-pen XL^®^ tonometer (Reichert, NY, United States) calibrated according to the manufacturer’s instructions. IOP was recorded until the animals (*n* = 6) were accustomed to the experimental procedure. Each formulation (50 μL) was instilled topically into the right eye, while the left eye received no treatment to minimize the diurnal and individual variations. The IOP was measured at 0.5, 1, 2, 3, 4, 5, 6, 8, 10, 12 and 24 h after instilled.

### Statistical Analysis

The data were expressed as mean ± SD. Statistical significance was assessed by a two-way analysis of variance using SPSS 16.0 (SPSS, Inc., Chicago, IL, United States). The *p*-values < 0.05 were considered statistically significant difference.

## Results

### UV/vis Spectroscopy

**FIGURE 2:**
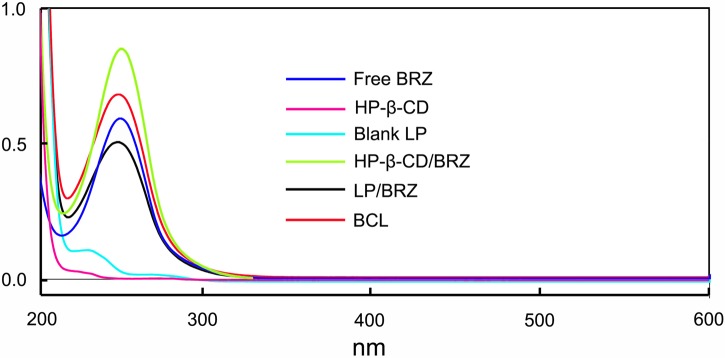
The UV/vis spectra of free BRZ, HP-β-CD, Blank LP, HP-β-CD/BRZ inclusion complex, LP/BRZ, and BCL in acetonitrile.

The influence of the BCL on the absorption of BRZ was evaluated by UV/vis spectroscopy. The absorption spectra of BRZ, HP-β-CD, Blank LP, HP-β-CD/BRZ inclusion complex, LP/BRZ and BCL in acetonitrile were determined (**Figure 2**). The spectrum of BCL exhibited a characteristics absorption peak at 252 nm, which was similar to the peak of BRZ. These results suggested that the HPLC method developed for BRZ measurement with a detection wavelength of 252 nm could also be applied to determine the BRZ concentration in BCL.

### Preparation of BCL

**FIGURE 3:**
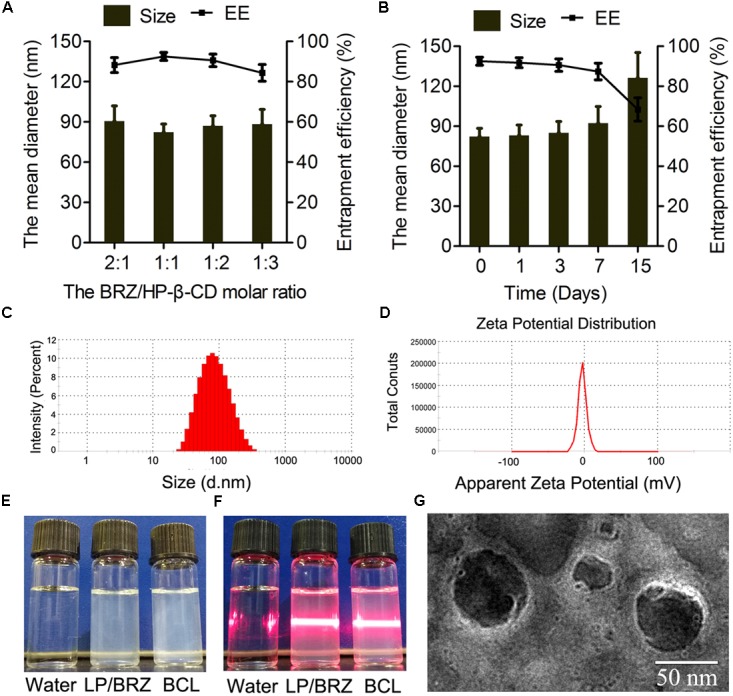
Effects of various HP-β-CD/BRZ ratios on diameter and drug entrapment efficiency of BCL **(A)** (*n* = 3). Change in size and entrapment efficiency of BCL at various time points stored at 4°C **(B)** (*n* = 3). Size distribution **(C)**, ζ potential **(D)**, appearance **(E)**, and tyndall effect **(F)** of BCL. TEM images of BCL **(G)**.

Different ratios of BRZ and HP-β-CD were used to establish the optimal preparation conditions in which BCL are formed. The drug content of BCL ranged between 84.38 and 92.50% and the size of BCL ranged between 82.29 ± 6.20 nm and 90.62 ± 11.30 nm depending on the BRZ/ HP-β-CD molar ratio (**Figure 3A**).

### Characterization of BCL

#### Particle Size, ζ Potential, and EE

The physicochemical properties of optimal BCL were evaluated. The particle size of the optimal BCL was 82.29 ± 6.20 nm with a narrow size distribution (PDI = 0.21 ± 0.01) (**Figure 3C**). The ζ potential of BCL was -3.57 ± 0.46 mV (**Figure 3D**). The appearance and morphological studies of BCL were also conducted. LP/BRZ and BCL colloidal solution were observed as a slightly blue opalescence with obvious Tyndall effect compared with water (**Figures 3E,F**). BCL were seen to be distinct lipid membrane structure with a particle size diameter of about 70 nm (**Figure 3G**).

#### Storage Stability

The particle size and EE of BCL were determined at a predetermined time of storage at 4°C. BCL displayed good stability with no detectable changes in particle size and EE for at least 3 days (**Figure 3B**).

#### XRD Analysis

**FIGURE 4:**
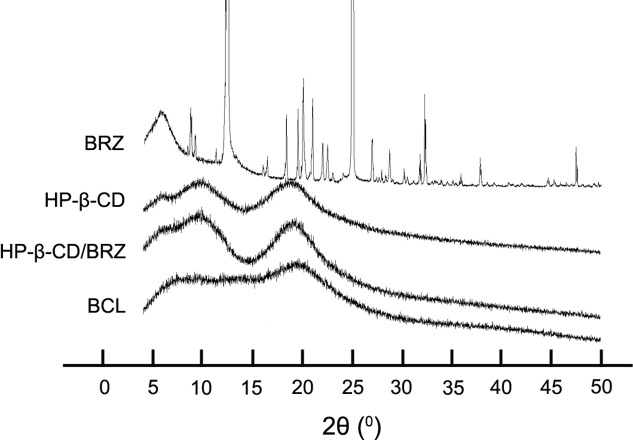
X-ray diffractograms of BRZ, HP-β-CD, HP-β-CD/BRZ, and BCL.

X-ray diffractograms were obtained from different samples of BRZ, HP-β-CD, HP-β-CD/BRZ and BCL to evaluate the crystal structure and entrapment of BRZ in BCL. The X-ray spectrum of BRZ showed several sharp and narrow peaks between 5 and 50°C (2θ) with a maximal peak at 2θ = 24.97°C, indicating their crystalline nature. HP-β-CD displayed a specific broad peak at 2θ = 20°C in its X-ray diffractogram. Neither HP-β-CD/BRZ nor BCL showed the peak at 2θ = 24.97°C (**Figure 4**).

#### *In Vitro* Release Profile

**FIGURE 5:**
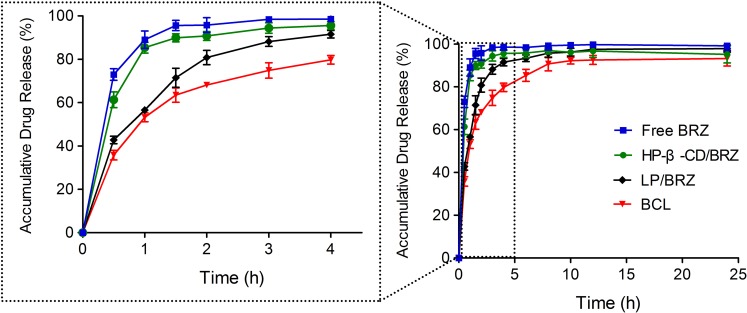
*In vitro* release profiles of BRZ from BRZ solution (free drug), HP-β-CD/BRZ, LP/BRZ, and BCL at 37°C in simulated tear fluid. Data are expressed as mean ± SD (*n* = 3).

The *in vitro* release experiments were performed to investigate the successful inclusion and the sustained release characteristic of BCL. A sustained release phase was observed in BCL within a period of 9 h (1–10 h) as presented in **Figure 5**. BRZ release from the inclusion complex was slightly slower than free drug but faster than LP/BRZ. Moreover, BCL displayed a slower release compared to LP/BRZ.

### Corneal Permeation and Corneal Hydration

**FIGURE 6:**
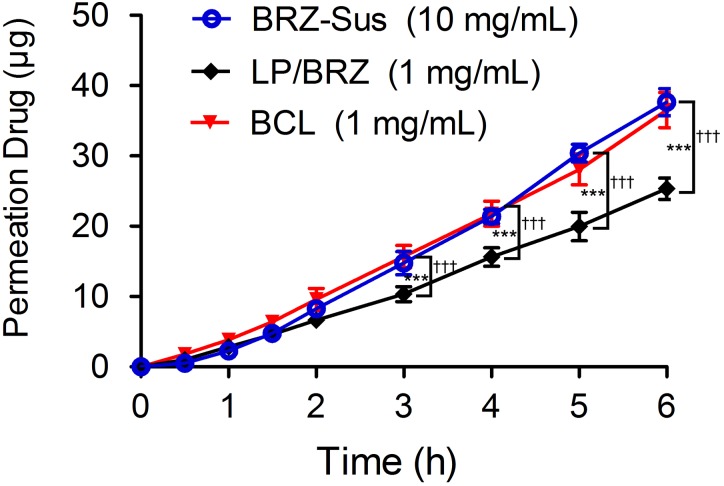
*In vitro* transcorneal permeation profiles of BRZ-Sus, LP/BRZ, and BCL versus time. Data are expressed as mean ± SD (*n* = 3) (^∗∗∗^*p* < 0.001 vs. BRZ-Sus; ^†††^*p* < 0.001 vs. BCL).

The corneal penetration study was performed to evaluate the effect of BCL on transcorneal transportation. The corneal permeation characteristics of BRZ-Sus (10 mg/mL), LP/BRZ (1 mg/mL), and BCL (1 mg/mL) were shown in **Figure 6**. There was no significant difference in the cumulative permeation amount at 0–2 h among the three groups. At 3–6 h, BRZ-Sus and BCL showed a significant increase of BRZ cumulative permeation compared with LP/BRZ. A linear relationship was observed for all preparations over the time period from 0.5 to 6 h, indicating that the corneal integrity was maintained throughout the experiment. Moreover, the permeation coefficient of BCL was much larger than those of BRZ-Sus and LP/BRZ (**Table 1**, *p* < 0.001). BCL showed a 9.36- and 0.40-fold increase in P_app_ when compared with Brz-Sus and LP/BRZ, respectively.

**Table 1:** Corneal hydration level and P_app_ of different formulations containing brinzolamide. Data are expressed as mean ± SD (*n* = 3).

Formulation	HL%	P_app_ (×10^6^ cm/s)
BRZ-Sus	80.12 ± 2.43	0.36 ± 0.02
LP/BRZ	79.37 ± 2.19	2.59 ± 0.07
BCL	78.56 ± 3.04	3.73 ± 0.13

Corneal HL was used to evaluate the safety of BCL (Vega et al., 2008). Its normal value is often from 75 to 80% (Vega et al., 2008). The corneal HL of BRZ-Sus, LP/BRZ, and BCL were

80.12 ± 2.43%, 79.37 ± 2.19%, and 78.56 ± 3.04%, respectively. No statistical difference in HL % was found (*p* > 0.05), indicating BCL might be safe for topical ocular use.

### *In Vivo* IOP-Lowering Effect

**FIGURE 7:**
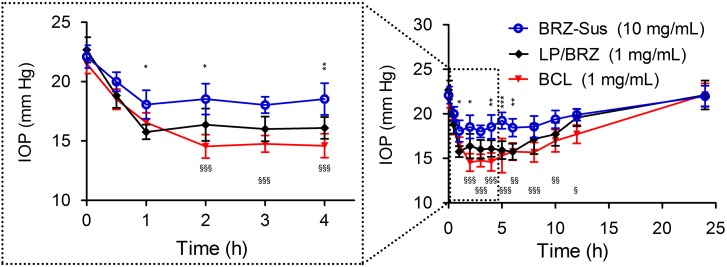
*In vivo* intraocular pressure (IOP) profiles of white New Zealand rabbits treated with BRZ-Sus, LP/BRZ, and BCL. Data are expressed as mean ± SD (*n* = 6) (^∗^*p* < 0.05, ^∗∗^*p* < 0.01, and ^∗∗∗^*p* < 0.001 vs. LP/BRZ; ^x^*p* < 0.05, ^xx^*p* < 0.01, and ^xxx^*p* < 0.001 vs. BCL).

As shown in **Figure 7**, BCL (1 mg/mL BRZ) presented more effective IOP reduction with a longer term role than BRZ-Sus (10 mg/mL Brz) and LP/BRZ (1 mg/mL Brz). The novel formulation of BRZ had an onset of less than 1 h, achieved a maximum IOP reduction of 32.3% at 2 h, and sustained an effective IOP reduction until the 12th hour, while Brz-Sus began to result in an effective IOP reduction at 0.5 h and quickly reached its peak effect at 1 h (an average reduction of 16.12% from baseline). Furthermore, IOP was significantly lower in the BCL group at any time point from 2 to 12 h than Brz-Sus group. Even though the dosage of BRZ was just 10% in BCL compared with BRZ-Sus, the IOP reduction efficacy was much higher.

## Discussion

In this work, we aimed to construct a novel ocular delivery system DCL loaded with BRZ (BCL) and investigate its potential to improve BRZ local glaucomatous therapeutic effect. BCL was prepared by hydrating lipid film with inclusion complex (Lapenda et al., 2013), and HP-β-CD was selected to enhance the solubility of poorly soluble drug BRZ (Zhang et al., 2013). According to the literature, the content of HP-β-CD was critical for the physicochemical properties of DCL (Chen et al., 2014; Cutrignelli et al., 2014). Cyclodextrins would interact with the lipid membrane and influence the formation of DCL (Cutrignelli et al., 2014). Thus, the amount of HP-β-CD was optimized based on the particle size and EE of BCL in this study. As seen in **Figure 3A**, EE first increased and then reached a plateau with the decrease of the BRZ/HP-β-CD molar ratio while the average diameter of BCL first decreased and then increased. Further increase in the HP-β-CD content (when the BRZ/HP-β-CD molar ratio was set at 1:3) cannot correspondingly enhance the BRZ entrapment into BCL but reduced EE. The cholesterol in the lipid bilayer of nanoliposomes might enter the excessive HP-β-CD cavity (Morrison et al., 2013; Chen et al., 2014), thereby forming unstable nanoliposomes which cannot incorporate enough HP-β-CD/BRZ-inclusion complex. The highest EE and the smallest particle size of BCL were achieved when BRZ and HP-β-CD was fed at a 1:1 molar ratio, and thus BCL containing 1:1 BRZ/H-β-CD were chosen for the subsequent investigations.

The optimal BCL were nano-sized (less than 100 nm) with negative surface charge probably resulting from the phosphatidylcholine head group in the outer nanoliposomes surface (Ascenso et al., 2013). Interestingly, BCL colloidal suspension had a similar appearance, average size, and surface charge to LP/BRZ despite the introduction of HP-β-CD (Maestrelli et al., 2010). This phenomenon indicated that HP-β-CD might mainly distribute in the inner aqueous core of the nanoliposomes, which was consistent with the literature (Cavalcanti et al., 2011; Lapenda et al., 2013; Chen et al., 2014). Moreover, it was clear that the peaks of BRZ disappeared from the diffraction pattern of BCL, demonstrating that BRZ was completely and successfully encapsulated into BCL.

BCL could be stable for about 7 days at 4°C, which might benefit from the negatively charged surface. However, EE sharply declined when BCL were kept for 15 days. The cholesterol in BCL might go into the hydrophobic cavity of HP-β-CD to compete with BRZ (Lapenda et al., 2013; Morrison et al., 2013), hence partially contributing to partial leakage of BRZ from the inclusion complex and the instability of nanoliposomes. HP-β-CD has previously been reported to extract cholesterol from cell membranes (Morrison et al., 2013) and decrease the integrity of liposomes composed of cholesterol and saturated phospholipids (Hatzi et al., 2007). The phenomenon found in this study was in line with these former studies. The free BRZ would easily pass through the lipid bilayer of the unstable BCL, which led to the reduced EE after relatively long-term storage. Generally, this kind of destructive role of HP-β-CD on DCL basically depends on the HP-β-CD affinity balance between the guest drug molecules and lipid components of nanoliposomes (Piel et al., 2006; Chen et al., 2014). According to the preliminary stability results, BCL would be further developed to be a freeze-drying formulation for long-term storage.

BCL performed a slower release than HP-β-CD/BRZ inclusion complex and LP/BRZ. It seems that the slower release profile of BCL was partially attributed to the fact that there were more barriers to the diffusion of BRZ from the BCL than LP/BRZ. As for BCL, two definitely different routes may account for the BRZ release (Chen et al., 2014). The whole HP-β-CD/BRZ inclusion complex might release from the destructive BCL or transport to lipid phase and then release. For the other way, BRZ might release from the dissociated HP-β-CD/BRZ inclusion complex inside BCL followed by diffusion out of the nanoliposomes and dispersion through the dialysis bag (Chen et al., 2014). To date, there still remains a lot of work to do to clarify the relative contributions of the two definitely different routes in release of BCL.

BCL exhibited enhanced and sustained IOP reduction effect as shown in **Figure 7**. This promising efficacy of BCL on glaucoma treatment may be mainly explained by the combinatorial advantages of BCL with HP-β-CD and nanoliposomes. Neutral liposome with a size of about 100 nm were reported to be safe for ocular applications without ocular irritability (Taniguchi et al., 1988). Moreover, particle size < 10 μm was recommended and considered optimal for ophthalmic preparation to minimize eye irritation (Kassem et al., 2007; Yuan et al., 2009). Accordingly, BCL colloidal solution with small size rather than BRZ-Sus might attenuate ocular irritation, thereby reducing the loss of BRZ by tear flushing. In addition, BCL with higher corneal binding affinity (Natarajan et al., 2012) can increase the retention on the surface of cornea, probably extending the absorption of BRZ. BCL displayed significant improvement in corneal permeation in contrast to the conventionally prepared LP/BRZ. This was thought to be the cause of the more efficient IOP lowering effect of BCL. Moreover, the intact HP-β-CD/BRZ inclusion complex released from BCL might lead to the enhanced penetration according to our previous study (Zhang et al., 2013). Of note, the sustained release profile of BCL as shown in the *in vitro* release study might be linked to the prolonged therapeutic efficacy of BCL.

## Conclusion

In this study, a novel drug-in-cyclodextrin-in-nanoliposome system containing BRZ was successfully prepared and its potential application in glaucomatous treatment was investigated. The optimized ratio of BRZ and HP-β-CD was set at 1:1 to prepare BCL with small size and high drug content. BCL incorporated BRZ very well and showed a sustained release profile and enhanced corneal permeation. Moreover, this new formulation of BRZ achieved an improved and prolonged effect of IOP reduction with similar safety to the commercially available formulation on white New Zealand rabbits. However, further studies are still required to investigate the *in vitro* release properties of BCL and elucidate how the release profile affects the therapeutic efficacy of BCL. In sum, all the data indicated that DCL might be one of the potential ocular delivery systems for hydrophobic drugs and BCL were worthy of further investigation as a novel anti-glaucoma formulation candidate of BRZ.

## Author Contributions

XS conceived of the study. JW designed the experiments. XS and FW wrote the manuscript. FW and XB conducted most of the experiments. AF performed the statistical analysis and drafted the manuscript. HL and YZ performed all the preliminary experiments. YL and CJ participated in literature research and manuscript editing. All authors reviewed and approved the manuscript.

## Conflict of Interest Statement

The authors declare that the research was conducted in the absence of any commercial or financial relationships that could be construed as a potential conflict of interest.
